# Differences in Morphology of Rural vs. Urban Individuals of the Flightless Ground Beetle, *Carabus convexus*

**DOI:** 10.3390/insects16040430

**Published:** 2025-04-19

**Authors:** Tibor Magura, Roland Horváth, Szabolcs Mizser, Mária Tóth, Gábor L. Lövei

**Affiliations:** 1 Department of Ecology, Faculty of Science and Technology, University of Debrecen, Egyetem Sq. 1, H-4032 Debrecen, Hungary; horvath.roland@science.unideb.hu (R.H.); mizser.szabolcs@science.unideb.hu (S.M.); toth.maria@science.unideb.hu (M.T.); 2 HUN-REN–UD Anthropocene Ecology Research Group, Egyetem Sq. 1, H-4032 Debrecen, Hungary; gabor.lovei@agro.au.dk; 3 Flakkebjerg Research Centre, Department of Agroecology, Aarhus University, DK-4200 Slagelse, Denmark

**Keywords:** adaptation, carabid, dispersal, fragmentation, habitat modification, home range, human disturbance, locomotory capacity, trait, urbanization

## Abstract

Urbanization causes significant environmental and structural changes in habitats, one of them being increased fragmentation. Traits associated with increased locomotory capacity may be advantageous in such situations, as individuals with those traits may expand their home range or have a chance to escape the patch where conditions threaten their survival. Individuals of the forest specialist, flightless ground beetle *Carabus convexus* in urban habitats may respond to urbanization by increasing their locomotory capacity (increased muscle mass) with respect to their conspecifics in rural habitats. In order to test this hypothesis, morphological traits standardized for body size were assessed using linear mixed-effects models. Pronotum volume (as a proxy for muscle mass) showed no significant difference between urban and rural individuals. The size of the tibia and femur of the front, middle, and hind legs (a proxy for leg muscle mass) significantly differed between sexes, with males having significantly larger tibiae and femora than females. Furthermore, urban males had significantly larger hind tibiae than rural conspecifics. Sex-specific differences in tibia and femur size is expected because males usually have higher locomotory activity than females. Larger tibiae of urban males can be advantageous to extend their home range, ensuring that males find mating partners even in low-density urban populations.

## Simple Summary

Urbanization leads to increased fragmentation when moving between different suitable fragments becomes important. This profound environmental change favors traits that improve movement ability. One of these is increased muscle mass that may help animals move more easily, expand their territory, or escape harmful changes. Dispersal capacity of flightless beetles is determined by functional leg muscles, and we hypothesized that adults of the flightless, forest specialist ground beetle *Carabus convexus* had more muscle mass in urban than rural areas. However, we found no significant difference in muscle mass (measured by pronotum volume) between urban and rural individuals. Urbanization-related differences in hind leg muscle mass (reflected by tibia and femur size) were found. Urban males had significantly larger muscle mass than rural ones, likely as an adaptation to cover larger areas in search of mates, possibly because of low-density urban populations.

## 1. Introduction

Global anthropogenic impacts, including forestry, agriculture, and urbanization, significantly affect biodiversity through habitat exploitation and habitat loss, as well as the alteration, fragmentation, and isolation of remaining habitat patches, fundamentally influencing ecosystem health, functions, and services [1,2,3]. Urbanization, characterized by the expansion of urban land use forms, the exponential increase in urban human population, and the spread of urban lifestyles, is one of the most significant element of anthropogenic global change [4,5,6].

Urbanization causes considerable changes in many environmental and habitat parameters. Artificial land surfaces related to industrial, commercial, administrative, residential, public, and transport infrastructures modify wind currents, strongly influencing the microclimate and creating the urban “heat island” [7,8,9]. Human activities, including industrial production, transport activity, and domestic operations trigger additional related changes in humidity [10], soil properties [11,12], and air, soil, and water pollution [13,14]. Urban changes in environmental parameters are often combined with degradation, fragmentation, and isolation [15] of habitats, as well as with community-wide shifts facilitated by invasion of exotic and generalist species [16]. These changes also affect biotic interactions [17], as well as ecological functions and processes, including decomposition [18], mineralization [19], predation [20], and pollination [21].

Several of these are ecological novelties [22] to which urban dwellers need to be able to adapt. Changes in behavior are perhaps the first and fastest responses to urban ecological novelties, and urban-living animals often become bolder and more exploratory. This was documented not only in vertebrates [23,24,25] but also invertebrates [26,27]. Traits related to condition and morphological parameters (e.g., body size, body mass) have direct fitness implications, affecting survival and reproductive success [28,29,30]. Other morphological traits, associated with increased dispersal power (e.g., longer/larger wings, larger legs, greater muscle mass), may also be beneficial in changing environments, as individuals with those traits may expand their home range or may disperse more easily between habitat fragments and escape from patches where environmental conditions have become unfavorable. Indeed, individuals living in urban habitats often have longer/larger wings [31,32,33] and/or larger legs [32,34]. Studies on urbanization-triggered changes in muscle mass are very few and biased toward birds [35] and flying insects [36]. Terrestrial arthropods have been less studied (but see [32]), and studies on flightless species are completely lacking, although well-developed muscles are clearly important for their dispersal.

Ground beetles (Coleoptera: Carabidae) are prominent members of the terrestrial arthropods and favorite subjects of urbanization studies because their well-known taxonomy and ecology, abundance and diversity, and the availability of appropriate and useful methodological tools [37]. As dispersal capacity of flightless ground beetles is determined entirely by the functional leg muscles [37,38], we hypothesized that environmental and habitat changes accompanying urbanization favor morphological traits related to increased dispersal power of urban beetles. More precisely, we assumed that urban individuals of our habitat specialist, flightless model ground beetle species should have increased locomotory capacity (increased muscle mass relative to body size) in urbanized environments with respect to their rural conspecifics.

In the present study, we found that the pronotum volume standardized for body size (as a proxy for muscle mass) was not significantly different between urban and rural individuals. However, urban males had significantly larger hind tibiae (a proxy for leg muscle mass, also standardized for body size) than rural conspecifics.

## 2. Materials and Methods

### 2.1. Study Area

Our study areas were parts of the Great Forest of Debrecen, on the eastern part of Hungary. This is an extensive lowland forest, >120 years old, dominated by English oak (*Quercus robur*). The forest is adjacent to the city to the north, part of the Natura 2000 network (site code: HUHN20033), but several fragmented and isolated patches of the once continuous forest are now within the city. These urban forest fragments are separated from the surrounding rural forest stands by several multi-lane asphalt-paved roads, negatively affecting immigration from rural areas [39]. The long-term Hungarian site of the international GLOBENET program that assesses the impacts of landscape changes on biodiversity [40] was established in this area. The program’s strict site selection criteria ensure that the main environmental parameters (e.g., groundwater levels, soil minerals, vegetation) were similar in rural and urban study sites. This situation provides an excellent opportunity to select sampling sites in identical forest associations with the same history, representing the two endpoints of an urbanization gradient. These conditions allow analysis of the various ecological effects of urbanization [41]. We sampled four rural and four urban forest fragments, defined by differences in the proportion of built-up land surfaces, trampling intensity, habitat management interventions, soil and soil surface temperature, soil pH, calcium (Ca) and zinc (Zn) concentrations in the soil, and the amount of decaying wood, as detailed in Magura et al. [42,43,44]. All studied sites were at least 3 ha and at least 250 m from each other (mean distance between the rural sites: 396.5 m; between urban ones: 702.2 m).

### 2.2. Model Species and Sampling

The chosen species, *Carabus convexus* F., 1775 is a medium-sized, flightless, predatory ground beetle that is widespread in Eurasia [45]. In the studied region (Great Hungarian Plain), *C. convexus* is a forest specialist species [46]. This species is very sensitive to urbanization-related environmental and habitat changes, as well as disturbances [47,48]. Thus, in the studied isolated urban forest fragments, its occurrence is sporadic; the density of surviving populations is significantly lower than in rural forest stands [49]. Paved roads absorb heat, and this, with high light intensity and lack of shelter on their surfaces, makes them dispersal barriers for flightless ground beetles, especially for forest specialists [50,51,52]. Therefore, the surviving *C. convexus* in the urban forest fragments have become completely isolated and exposed to population-level effects of fragmentation and isolation for multiple generations.

Individuals of *C. convexus* were collected using 15 live capture traps per site, arranged as in Magura et al. [27,43]. Sampling was performed from April to June and from September to October in 2020, excluding the summer aestivation period [37]. Traps were controlled twice per week, and sampled beetles (69 from rural and 13 from urban sites) were transported to the laboratory.

### 2.3. Evaluating and Measuring Morphological Traits

In the laboratory, species identity was confirmed using standard keys [53], and their sex established. Individuals were placed into micro centrifuge tubes (2 mL), euthanized by freezing, and stored at −17 °C. Individual elytra length was used as a proxy for body size [54]. Before measuring them, beetles were defrosted, and individually placed in a standard, horizontal position in a plastic Petri dish filled with small (diameter 2 mm) glass beads, with a microscope calibration slide (0.1 mm precision) [55]. Beetles were photographed using a digital camera (Olympus C7070WZ, Olympus Corporation, Tokyo, Japan) mounted on a stereomicroscope (Olympus SZX7). Elytral length (from the lower end of the scutellum to the apex of the left elytron) was measured with Adobe Photoshop (CS6, version 13.0.1) with a precision of 0.001 mm. The mean of triplicate measurements was used as proxy for body size.

Pronotum dimensions (e.g., width) are relevant proxies for movement ability [56] because the pronotum bears muscles and supports the locomotion of the prothoracic (front) legs [57]. We used pronotum volume as a proxy for locomotor muscle mass. Pronotums were carefully removed and photographed both dorsally and laterally using the same method detailed above. From these photos, the width, height, and depth of the pronotum were measured as above. The pronotum volume was calculated as the product of the pronotum width, height, and depth. Tibia and femur area were used as proxies for leg muscle masses because they contain important intrinsic leg muscles [57]. The tibia and femur of the front, middle, and hind leg from the left-hand side of each beetle were carefully removed and mounted upon microscope slides and photographed, and the area of the tibia and femur was calculated with Adobe Photoshop. The mean of triplicate measurements was used for analyses.

### 2.4. Statistical Analyses

All statistical analyses were performed in the R program environment (version 4.4.1 [58]). Elytral length (as a proxy for body size) was not significantly different between rural and urban beetles, but all studied morphological traits related to locomotory ability (pronotum volume; tibia area and femur area of the front, middle, and hind leg) significantly depended on body size (Tables S1 and S2, Figures S1–S7). Therefore, to avoid the potential bias of simply detecting changes in the studied morphological traits because of changes in body size, all morphological trait values were standardized for body size by dividing them by the elytral length.

Differences in the morphological traits were tested using linear mixed-effects models (LMMs) using the *lme4* package [59]. Before modelling, the probability distribution that best fitted the response variable was examined using the *car* [60] and the *MASS* packages [61]. Based on these analyses, all response variables (standardized pronotum volume; tibia area and femur area of the front, middle, and hind legs) were modelled using a normal error distribution. The fixed effects in the models were the urbanization level (rural vs. urban), sex (female vs. male), and their interaction. The nested sampling design (sites nested within areas) was included as a random effect as well as the sampling month. As one of our random effects was nested, the parameters in the mixed models were estimated using the maximum likelihood method [62]. In case of a significant difference between the means, the Tukey test with unequal sample sizes was performed using the *agricolae* package [63]. The small sample size in the urban area may considerably limit the statistical power of the models, thereby increasing the risk of failing to detect differences even if they exist (Type II errors). Therefore, the statistical power of the models was assessed using the *simr* package [64]. First, we assessed the statistical power of our linear mixed-effects models to detect biologically meaningful effect sizes. Specifically, we tested whether a 1–20% difference in our response variables between urban and rural beetles could be detected with sufficient power (at α = 0.05) using our original sample size. Furthermore, we tested by simulation the effect of increased sample size (the number of females and males tested in rural and urban areas) on the statistical power of the models to detect significant differences.

## 3. Results

Eighty-two *C. convexus* individuals were caught in the studied sites, including 69 (35 females and 34 males) at the rural sites and 13 (8 females and 5 males) at the urban ones. All beetles had sharp or very little worn mandibles, indicating that they were in their first breeding season.

**Figure 1:**
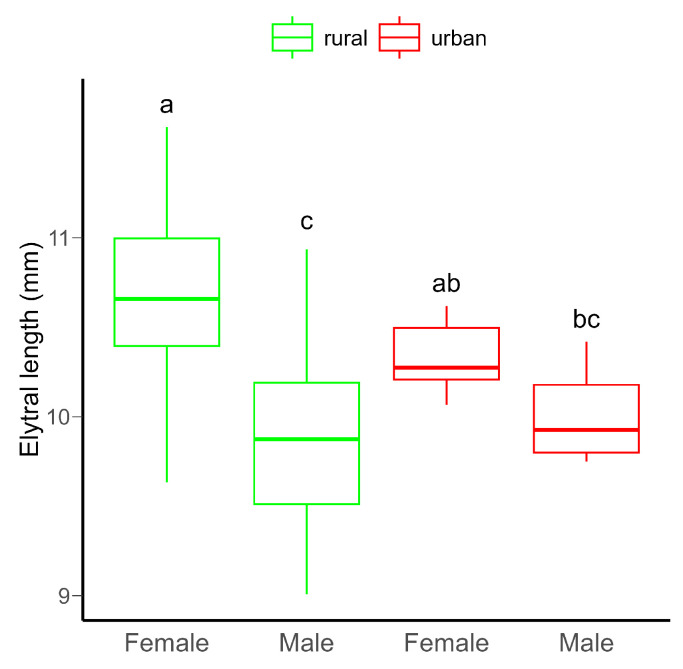
Boxplot of the elytral length (as proxy for body size) of *C. convexus* beetles sampled in rural and urban habitats. In boxplots, the horizontal lines represent median values, the boxes denote interquartile ranges, while whiskers show minimum and maximum values. Different letters indicate significant (*p* < 0.05) differences based on the Tukey test.

Although no significant difference in body size between rural and urban beetles was detected, females were significantly bigger than males (Table S1, Figure 1). Furthermore, there was a significant variation in body size between males and females in both habitats (Table S3).

Simulations indicated that with our sample size, 2.5–5% differences in response variables between the rural and urban beetles could be detected with 80% power at α = 0.05, except for the pronotum volume (Figure S8). However, the differences estimated using the linear mixed-effects models are much smaller (Table 1), so sufficient power to detect existing differences would probably be obtained by increasing the sample size.

**Figure 2:**
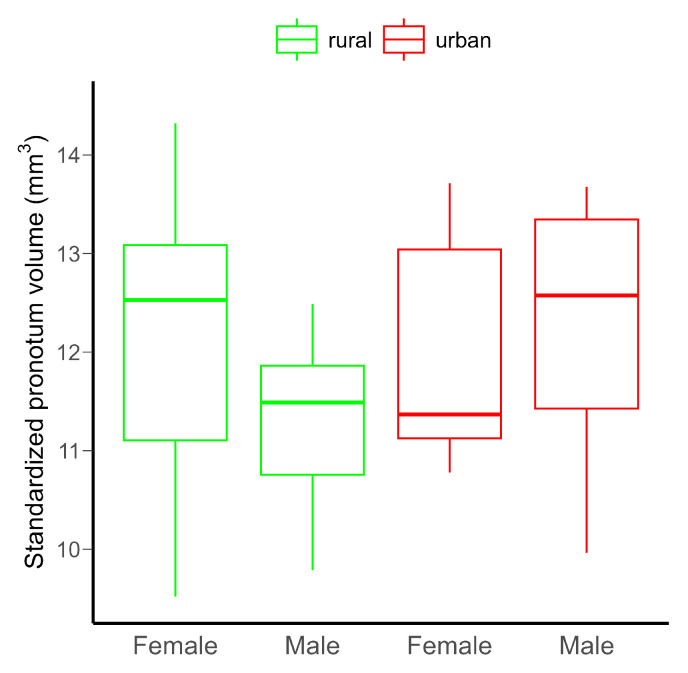
Differences in the standardized pronotum volume in *C. convexus* adults in rural and urban habitats. The horizontal lines indicate medians, and the boxes the interquartile ranges, with whiskers showing minimum and maximum values. There were no significant (*p* < 0.05) differences among groups.

Neither the urbanization level (rural vs. urban), the sex of the beetles (females vs. males), nor their interaction had detectable significant effect on the pronotum volume, probably due to the low statistical power of the model (Table 1 and Figure 2). However, power would not be increased by increasing the sample size (Figure S9).

**Figure 3:**
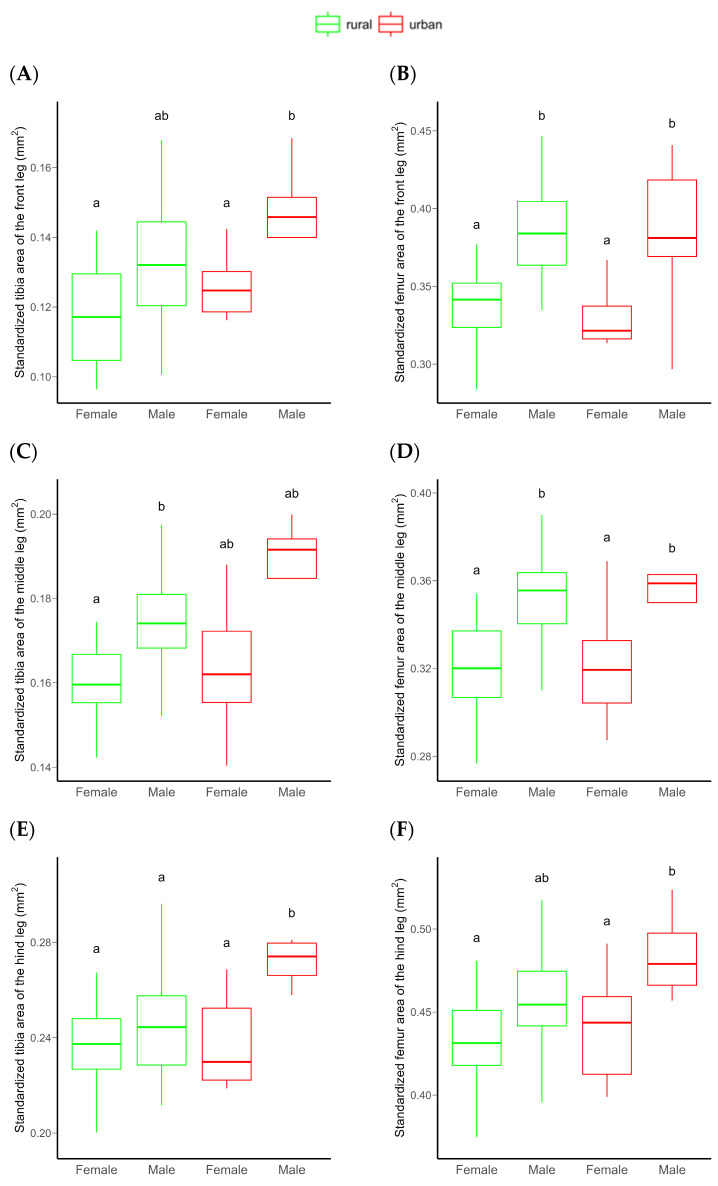
Differences in the standardized frontal (**A**,**B**), middle (**C**,**D**), and hind (**E**,**F**) tibial and femoral area of *C. convexus* beetles sampled in rural and urban habitats. In boxplots, the horizontal lines represent median values, the boxes denote interquartile ranges, while whiskers show minimum and maximum values. Different letters indicate significant (*p* < 0.05) differences based on the Tukey test.

**Figure 4:**
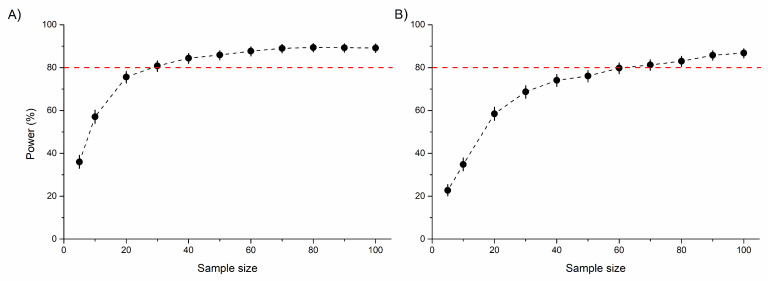
Statistical power (%) of linear mixed-effects models to detect the model-estimated differences (2.15% for tibia and 3.06% for femur) in the standardized tibia (**A**) and femur (**B**) area of the hind leg between rural and urban beetles. Sample size represents the number of beetles per sex (female or male) per area (rural or urban). Red dotted lines indicate the 80% power limit.

The area of both the frontal tibia and femur were significantly higher in males than females. However, no significant differences were detected between rural vs. urban individuals or for the urbanization level × sex interaction, probably due to the low statistical power of the model (Table 1 and Figure 3A,B). An increasing sample size would not substantially improve this relationship (Figure S9). Similarly, the tibia and femur area of the middle leg (a proxy for leg muscle mass) significantly differed between sexes, with males having significantly larger tibiae and femora than females. For these morphological traits, no significant habitat-related difference was detected, possibly due to the low statistical power of the model (Table 1 and Figure 3C,D). This power did not increase significantly by increasing the sample size (Figure S9). Both the standardized tibia area and the standardized femur area of the hind leg (as proxies for leg muscle masses) were also significantly higher in male than in female beetles. In this case, the urbanization level (rural vs. urban) had a marginally significant effect on both. Furthermore, the interaction of urbanization level and sex also had a marginally significant effect on the tibial area. This effect was derived from the significantly larger hind tibiae of urban than rural males (Table 1 and Figure 3E,F). The statistical power of these models were moderate (Table 1), but expected to increase with increasing sample size (Figure 4).

## 4. Discussion

### 4.1. Methodological Considerations

In spite of the large trapping efforts, the sample size in urban forest fragments was small because the target species, *C. convexus*, is a forest specialist, and only a small population survived the consequences of urbanization [49]. No other species with similar characteristics was collected. This sample size limited the statistical power of the models with the consequent higher probability of Type II errors (failing to detect differences even if they exist). Indeed, for all tested morphological parameters, the statistical power of the model was below the generally accepted limit (80%). By increasing the sample size through simulation, the statistical power of the models remained largely unchanged for most traits. This suggests that urbanization-driven environmental changes are likely to have only very weak effects on these characteristics. Therefore, the observed non-significant differences in these traits were unlikely to be a result of small sample size. An alternative explanation could be that these morphological traits are not the most appropriate proxies for characterizing dispersal capacity. As the pronotum contains many different muscles (muscles for movement of the head, wing, and front leg [57]), its total volume is not necessarily a most reliable proxy for the locomotor muscle mass. The first two legs mainly pull, while the hind legs are responsible for the very powerful pushing movement [57]. Consequently, the muscle mass of the hind legs may be more important for dispersal. For the hind leg, the statistical power of the models increased with sample size, indicating that urbanization may indeed influence these traits, and the marginally significant effects were likely due to the limited sample size.

Although the muscles in the tibia only maintain stability during movement [57], our results suggest that the area of the hind tibia and femur is a sensitive proxy for dispersion capacity. Coxal and trochanter muscles (not studied in our study) are also important during movement [57]; therefore, their volume could be potential alternative parameters. Based on the results of our simulation analyses, we recommend that studies examining morphological traits related to dispersal capacity include a minimum of 40 individuals per habitat (both males and females) in order to obtain sufficient statistical power.

### 4.2. Evaluating Morphological Traits

Morphological traits, which significantly influence fitness, reproduction success, and ultimately survival, are frequently used as proxies to evaluate the impact of anthropogenic disturbances on animals. Changes in these traits can be associated with pollution [65,66] or changes in habitat conditions [67,68]. Among insects, body size is one of the most extensively studied morphological traits in the context of urbanization [69]. For ground beetles, body size exhibits considerable variation and is fundamentally linked to key biological attributes, including reproductive rate, home range, life cycle, population density, dispersal ability, resource utilization, and responses to environmental changes [37,70]. Larger species are generally more susceptible to anthropogenic impacts [29,71], consistent with the increasing disturbance hypothesis [72]. The most common metrics for expressing the body size of ground beetles are body mass and body length, which are closely related [73]. However, body mass can substantially fluctuate due to individual conditions (e.g., starvation, gonadal changes), while the size of the exoskeleton does not change, making it more suitable for quantifying the impact of anthropogenic disturbances [54,74]. Exoskeletal parameters used include total body length [75,76], elytral length [77,78], pronotum width [56,79], pronotum length [54,80], tibia length [34,56], and femur length [56,81], all of which exhibit significant variation within populations. These intrapopulation variations may arise from, or be influenced by, micro-environmental and individual factors. The immature life stages of ground beetles (eggs, larvae, and pupae) are particularly sensitive to microhabitat conditions due to their limited mobility. In particular, the soil-bound larvae are highly sensitive to environmental extremes and food shortages due to their weak chitinization and limited mobility. Larval feeding conditions often determine adult size, with consequences for fitness and fertility [37]. Consequently, oviposition site selection by females is crucially important [37,82]. Microhabitat- and individual sensitivity-driven variation in offspring morphology leads to noticeably differences in adult body size [37,82]. Indeed, the body sizes of both males and females varied significantly across our rural and urban sites. However, no significant differences in body size were observed between habitats for either sex. All examined morphological traits exhibited significant correlations with body size, but with unexplained variability. This unexplained variance may suggest that elytral length is not the most accurate proxy for body size, as significant, not size-related divergences in pronotum shape may exist between sexes of *Carabus* species [83], affecting the actual body size. Unexplained variance may arise from other parameters (e.g., body width, shape) that can also influence the values of morphological traits examined. Overall, to reduce the unpredictable influence of temporal microhabitat heterogeneity and individual sensitivity, it is advisable to standardize morphological traits for body size in studies on the effects of anthropogenic disturbances on morphology. When characterizing the body condition of ground beetles, correcting for body size is an established practice [78,81,84]. Such standardization is strongly recommended when examining morphological characteristics.

### 4.3. Sex-Specific Differences in Morphological Traits

Using tibia and femur areas standardized for body size as proxies for leg muscle masses—and thereby as indicators of movement/locomotory ability—in our flightless ground beetle species, we observed sex-specific differences. The movement ability of male beetles was significantly greater than that of females. Such sex-based disparities in movement ability may result from fundamental differences in reproductive investment strategies and behavior between the sexes. Female ground beetles allocate resources toward egg production to maximize their reproductive success. In contrast, males focus on maximizing mating opportunities to increase their reproductive success [37,56], which generally makes them more active than females [85,86]. Life-history theory predicts that such differences in reproductive investment strategies should result in pronounced sex-specific behavioral differences [87]. Supporting this, males of *Carabus nemoralis* O. F. Müller, 1764 are significantly more active and exploratory than females [88]. These behavioral differences likely contribute to the enhanced movement ability observed in males.

### 4.4. Urbanization-Related Differences in Morphological Traits

Our proxy measure indicated higher muscle mass in urban *C. convexus* males which allows higher mobility. Increased mobility is beneficial in fragmented environments, enabling individuals to expand their home ranges, disperse more easily between suitable habitats, and escape from patches with unfavorable environmental conditions. Processes and disturbances associated with urbanization—such as chemical, light, and noise pollution; the urban heat island effect; habitat alteration and loss; fragmentation and isolation; intensive management practices; and the invasion of exotic species—generally decrease the availability of suitable microsites for oviposition, overwintering, resting, and food [4,5,69]. Moreover, both intra- and interspecific competition for these spatially scattered resources can become more intense [89]. Additionally, aggregation of organisms in microsites with available resources can increase predation risk. Individuals with enhanced mobility are at an advantage when seeking spatially dispersed resources, and these individuals can reduce competition and minimize predation risk [3]. The size of home range in *Carabus* species is not precisely known. Movement distances (which are highly non-linear) range between 55 and 170 m over three weeks [90,91]. The average area of our urban forest fragments was 3.6 hectares. Even assuming a regular, circular shape, the minimum diameter of our urban forest patches was 1.26–3.9 times larger than the above three-week walking distance. This suggests that increased mobility to expand home range within a habitat patch may also be beneficial in urban habitat fragments. Increased mobility also supports metapopulation dynamics, ensuring the long-term survival of organisms in urban environments [92,93,94]. Additionally, urbanization often causes sudden and unpredictable changes in the local environmental conditions of habitat patches (e.g., temperature, humidity, pollution levels) that may exceed the tolerance limits of their inhabitants [5,7]. More mobile organisms can more easily escape from such unfavorable patches [95,96]. Our finding that only males exhibited a significant urbanization-related difference in the studied mobility-related traits may indicate sexual selection. In scramble competition for mating partners, greater mobility is likely advantageous for males. This mating strategy is widespread across animal taxa, including ground beetles [97]. Selection for increased mobility to increase mating success may be more intense in populations with low densities and limited availability of mates [97]. The population density of our model species, *C. convexus*, is significantly lower in the isolated urban forest fragments than in rural habitats [43,49]. As a result, urban males may be subject to selection for greater movement capacity [98]. Similar differences in movement capacity exist in males of another flightless ground beetle, *Carabus hortensis* L., 1758, in low-density populations at the edge of its range [56]. Individuals of higher mobility may exposed to higher predation risk [37,99] with a possible trade-off between reproductive success and predation risk. However, research on this topic remains limited, especially in insects, highlighting the need for further studies across diverse taxa to deepen our understanding.

## 5. Conclusions

Exploring differences in morphological traits related to locomotory ability is a key focus in ecology, as these traits influence survival, development, and reproduction, thereby directly shaping ecological population dynamics [100]. By comparing various morphological traits related to locomotory ability in a forest specialist, flightless ground beetle (*C. convexus*) from rural forest stands and urban forest fragments, we observed significant morphological differences between the sexes. Males exhibited significantly larger tibiae and femora (proxies for leg muscle mass) than females. These sex-dependent discrepancies in movement capacity likely arise from differences in reproductive investment and behavior. Male ground beetles actively search for females to maximize mating opportunities and, consequently, their reproductive success [37]. Additionally, urban males demonstrated significantly greater movement capacity (quantified by hind leg tibia size as a proxy for leg muscle mass) than their rural conspecifics. The enhanced movement capacity of urban males likely serves as an advantageous adaptation, enabling them to expand their home ranges and disperse more effectively across habitat fragments [94]. Moreover, greater mobility is advantageous for increasing mating success, particularly in low-density urban populations with limited mate availability [97]. Tracking individual movements using radio telemetry would be useful to document the increased movement activity of ground beetles in fragmented urban habitats [91]. Urbanization usually increases fragmentation of the original habitats, increasing the importance of successful dispersal by the remaining species among the remaining fragments in order to maintain self-supporting populations. Consequently, urban management practices aimed at enhancing connectivity between habitat patches are essential to preserving and maintaining urban biodiversity over time [41,69,92].

## Figures and Tables

**Table 1:** Summary of linear mixed-effects models and power analyses on morphological traits standardized for body size related to locomotory ability of rural vs. urban *C. convexus* adults (*p*-values in bold denote significant effects (*p* < 0.05), while italicized ones are marginally significant (*p* < 0.1)).

Response Variable	Fixed Effect	Estimate ± SE	χ^2^	df	*p*
Pronotum volume	Urbanization level	−0.0172 ± 0.8522	0.0004	1	0.9839
Statistical power: 8.70% (CI_95_: 7.03, 10.62)	Sex	0.5867 ± 0.3676	2.5476	1	0.1105
	Urbanization level × Sex	−0.2040 ± 0.9323	0.0479	1	0.8268
Area of the frontal tibia	Urbanization level	0.0112 ± 0.0080	1.9534	1	0.1622
Statistical power: 21.50% (CI_95_: 18.99, 24.18)	Sex	−0.0136 ± 0.0040	11.4015	1	**0.0007**
	Urbanization level × Sex	−0.0072 ± 0.0104	0.4750	1	0.4907
Area of the frontal femur	Urbanization level	−0.0024 ± 0.0133	0.0331	1	0.8557
Statistical power: 8.30% (CI_95_: 6.66, 10.19)	Sex	−0.0476 ± 0.0066	51.6991	1	**<0.0001**
	Urbanization level × Sex	−0.0038 ± 0.0173	0.0476	1	0.8273
Area of the middle tibia	Urbanization level	−0.0037 ± 0.0068	0.2890	1	0.5909
Statistical power: 14.90% (CI_95_: 12.75, 17.26)	Sex	−0.0182 ± 0.0035	27.7876	1	**<0.0001**
	Urbanization level × Sex	0.0084 ± 0.0088	0.9148	1	0.3388
Area of the middle femur	Urbanization level	−0.0029 ± 0.0110	0.0671	1	0.7955
Statistical power: 6.00% (CI_95_: 4.61, 7.66)	Sex	−0.0372 ± 0.0055	45.4003	1	**<0.0001**
	Urbanization level × Sex	0.0043 ± 0.0141	0.0933	1	0.7600
Area of the hind tibia	Urbanization level	0.0215 ± 0.0117	3.3710	1	***0.0664***
Statistical power: 50.70% (CI_95_: 47.55, 53.84)	Sex	−0.0128 ± 0.0045	8.0972	1	**0.0044**
	Urbanization level × Sex	−0.0207 ± 0.0122	2.8753	1	***0.0899***
Area of the hind femur	Urbanization level	0.0306 ± 0.0171	3.1998	1	***0.0736***
Statistical power: 42.40% (CI_95_: 39.31, 45.53)	Sex	−0.0228 ± 0.0078	8.5564	1	**0.0034**
	Urbanization level × Sex	−0.0210 ± 0.0213	0.9775	1	0.3228

## Data Availability

Data used for analyses are available in the Mendeley repository (doi: 10.17632/m4jcxmtc92.1; https://data.mendeley.com/datasets/m4jcxmtc92/1; accessed on 6 January 2025).

## Supplementary Materials

The following supporting information can be downloaded at: https://www.mdpi.com/article/10.3390/insects16040430/s1, Table S1. Summary of the linear mixed-effects model results on elytral length of *C. convexus* adults from rural vs. urban forested habitats (*p*-values in bold denote significant (*p* < 0.05) effects). Table S2. Summary of linear regression on the relationship between the elytral length (as a proxy for body size) of *C. convexus* adults and the studied morphological traits related to locomotory ability (*p*-values in bold denote significant (*p* < 0.05) relationships). Table S3. Summary statistics on elytral length (mm) of rural and urban adults of *C. convexus*. Figure S1. The relationship between the elytral length and pronotum volume in *C. convexus* adults collected in forested rural vs. urban habitats. The dashed red line is the fitted linear regression line (for detailed results, see Table S2). Figure S2. The relationship between the elytral length and the area of the frontal tibia in *C. convexus* adults collected in forested rural vs. urban habitats. The dashed red line is the fitted linear regression line (for detailed results, see Table S2). Figure S3. The relationship between the elytral length and the femur area of the front leg in *C. convexus* adults collected in forested rural vs. urban habitats. The dashed red line is the fitted linear regression line (for detailed results, see Table S2). Figure S4. The relationship between the elytral length and the area of the middle tibia in *C. convexus* adults collected in forested rural vs. urban habitats. The dashed red line is the fitted linear regression line (for detailed results see Table S2). Figure S5. The relationship between the elytral length and the femur area of the middle leg in *C. convexus* adults collected in forested rural vs. urban habitats. The dashed red line is the fitted linear regression line (for detailed results see Table S2). Figure S6. The relationship between the elytral length and the area of the hind tibia in *C. convexus* adults collected in forested rural vs. urban habitats. The dashed red line is the fitted linear regression line (for detailed results, see Table S2). Figure S7. The relationship between the elytral length and the femur area of the hind leg in *C. convexus* adults collected in forested rural vs. urban habitats. The dashed red line is the fitted linear regression line (for detailed results, see Table S2). Figure S8. The statistical power (at α = 0.05) of the linear mixed-effects models to detect 1–20% difference in the standardized pronotum volume (A), the standardized tibia and femur area of the front (B,C), middle (D,E), and hind legs (F,G) between urban and rural beetles with the original sample size (69 rural and 13 urban beetles). Red dotted lines indicate the generally accepted sufficient power limit (80%). Figure S9. Statistical power (%) of linear mixed-effects models to detect the model-estimated differences in the standardized pronotum volume (A) and the standardized tibia and femur area of the front (B,C) and middle legs (D,E) between rural and urban beetles. Sample size represents the number of beetles per sex (female or male) per area (rural or urban). Red dotted lines indicate the generally accepted sufficient power limit (80%).
