# Supplementary material for: Differences in Morphology of Rural vs. Urban Individuals of the Flightless Ground Beetle, Carabus convexus
Source: Insects. 2025 Apr 19;16(4):430. doi: 10.3390/insects16040430 (PMC12027500; doi:10.3390/insects16040430)
Supplement: Supplementary file 1 [file insects-16-00430-s001.zip › insects-3456072-supplementary.pdf]

Supplementary material to the article

Insects

Table S1. Summary of the linear mixed-effects model results on elytral length of *C. convexus* adults from rural vs. urban forested habitats (*p*-values in bold denote significant (p<0.05) effects).

Response Variable	Fixed Effect	Estimate ± SE	χ^2	df	*p*
Elytral length	Urbanization level	0.09419 ± 0.31613	0.0888	1	0.7657
	Sex	0.90045 ± 0.13258	46.1267	1	**< 0.0001**
	Urbanization level × Sex	-0.24832 ± 0.33724	0.5422	1	0.4615

Table S2. Summary of linear regression on the relationship between the elytral length (as a proxy for body size) of *C. convexus* adults and the studied morphological traits related to locomotory ability (*p*-values in bold denote significant (p<0.05) relationships).

Response Variable	Estimate ± SE	Adjusted R^2	F	df	*p*
Pronotum volume	23.375 ± 2.151	0.5942	118.10	1, 79	**< 0.0001**
Tibia area of the front leg	0.1288 ± 0.0296	0.1869	18.93	1, 77	**< 0.0001**
Femur area of the front leg	0.2407 ± 0.0556	0.1832	18.72	1, 78	**< 0.0001**
Tibia area of the middle leg	0.1596 ± 0.0271	0.2973	34.84	1, 79	**< 0.0001**
Femur area of the middle leg	0.2641 ± 0.0475	0.2721	30.91	1, 79	**< 0.0001**
Tibia area of the hind leg	0.2995 ± 0.0329	0.5059	82.92	1, 79	**< 0.0001**
Femur area of the hind leg	0.4659 ± 0.0557	0.4633	70.07	1,79	**< 0.0001**

Table S3. Summary statistics on elytral length (mm) of rural and urban adults of *C. convexus*.

Habitat, sex	Mean	S.D.	Minimum	Maximum
Rural females	10.7457	0.5627	9.6340	12.1347
Rural males	9.8299	0.6169	8.3733	11.4447
Urban females	10.0148	0.2805	9.7497	10.4193
Urban males	10.6285	0.7042	10.0667	12.1693

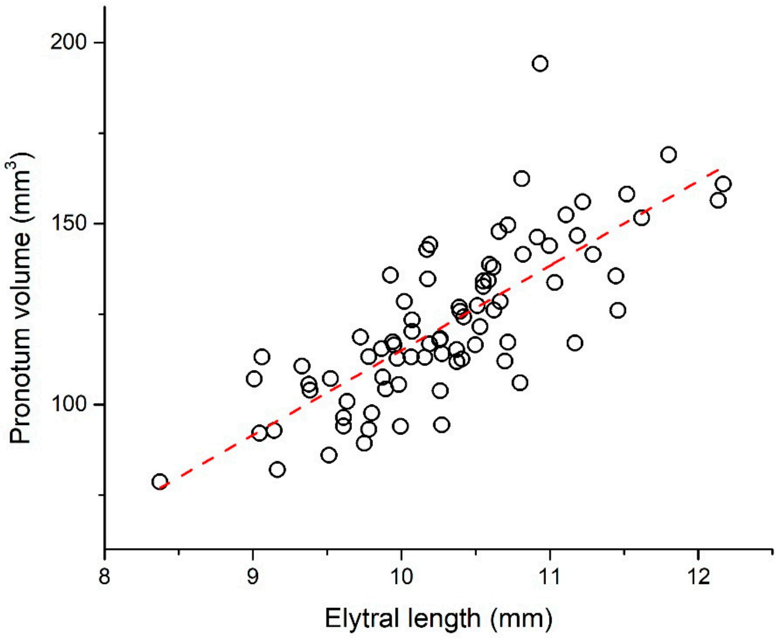

Figure S1. The relationship between the elytral length and pronotum volume in *C. convexus* adults collected in forested rural vs. urban habitats. The dashed red line is the fitted linear regression line (for detailed results, see Table S2).

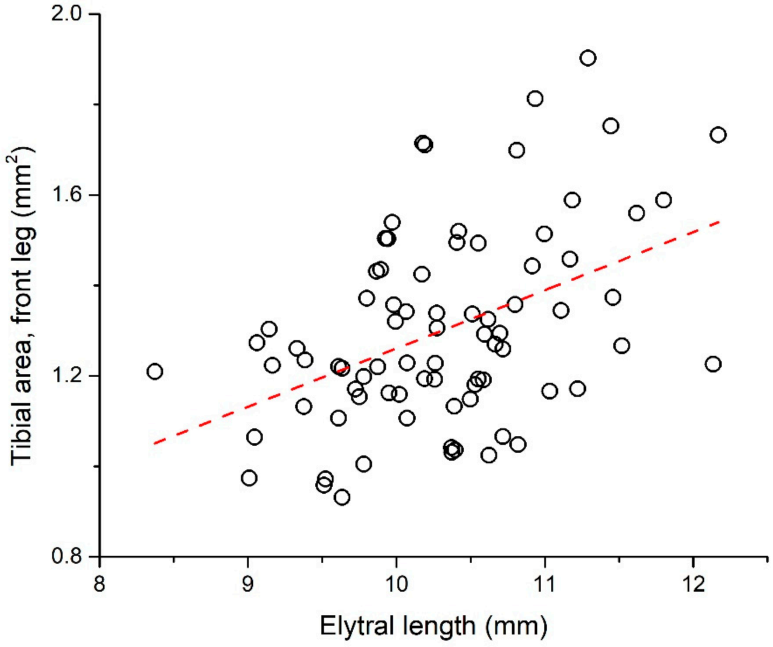

Figure S2. The relationship between the elytral length and the area of the frontal tibia in *C. convexus* adults collected in forested rural vs. urban habitats. The dashed red line is the fitted linear regression line (for detailed results, see Table S2).

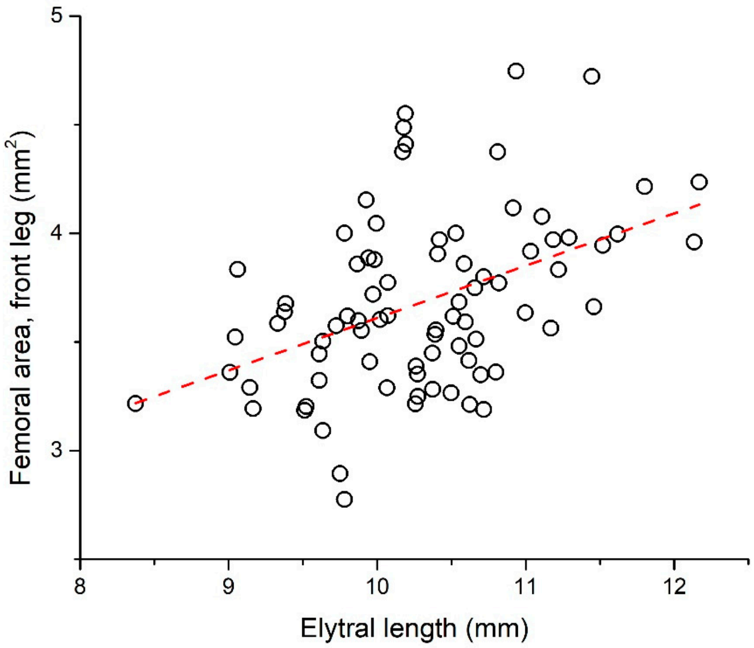

Figure S3. The relationship between the elytral length and the femur area of the front leg in *C. convexus* adults collected in forested rural vs. urban habitats. The dashed red line is the fitted linear regression line (for detailed results, see Table S2).

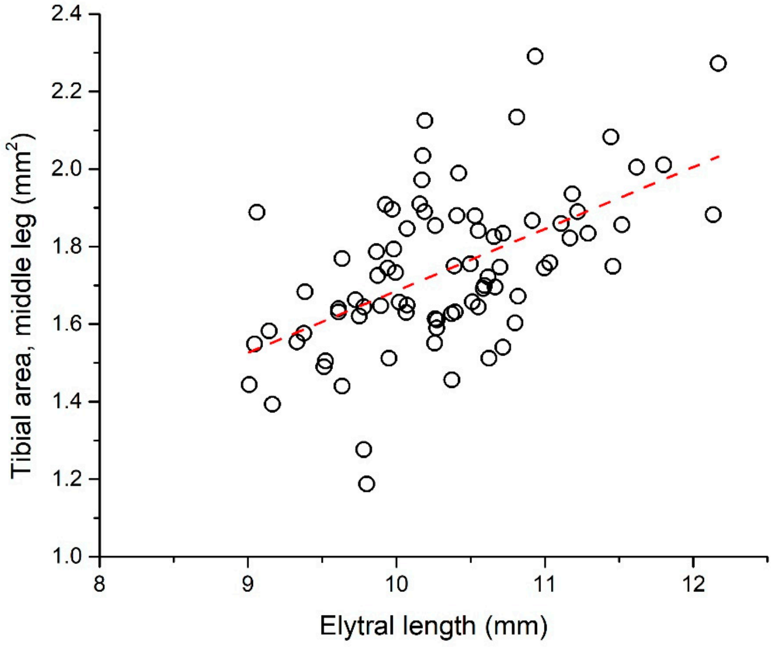

Figure S4. The relationship between the elytral length and the area of the middle tibia in *C. convexus* adults collected in forested rural vs. urban habitats. The dashed red line is the fitted linear regression line (for detailed results, see Table S2).

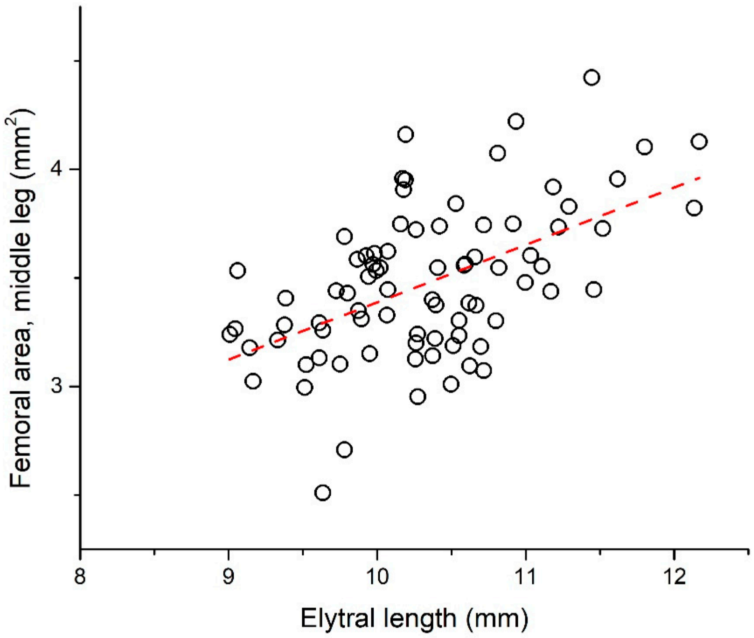

Figure S5. The relationship between the elytral length and the femur area of the middle leg in *C. convexus* adults collected in forested rural vs. urban habitats. The dashed red line is the fitted linear regression line (for detailed results, see Table S2).

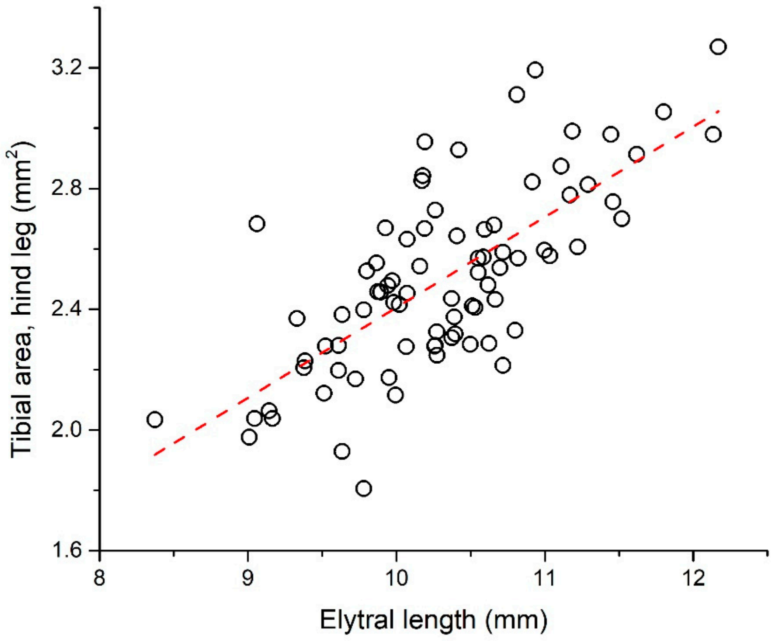

Figure S6. The relationship between the elytral length and the area of the hind tibia in *C. convexus* adults collected in forested rural vs. urban habitats. The dashed red line is the fitted linear regression line (for detailed results, see Table S2).

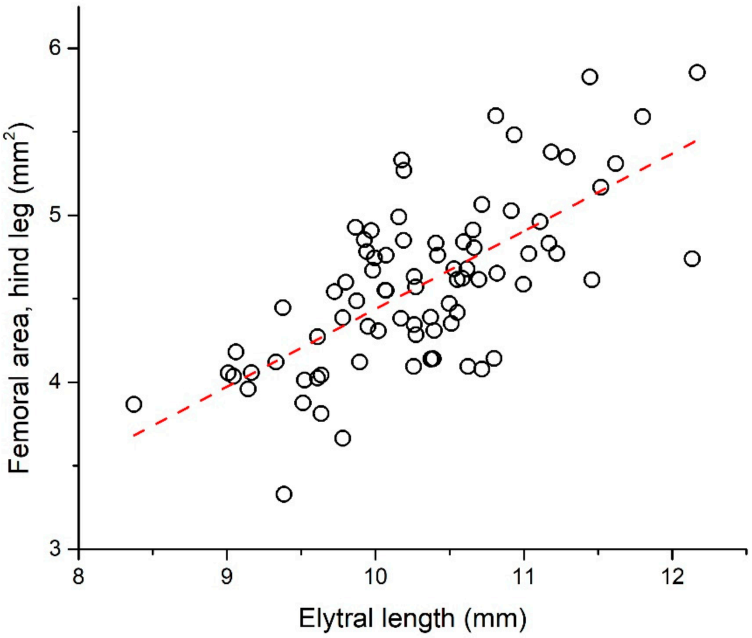

Figure S7. The relationship between the elytral length and the femur area of the hind leg in *C. convexus* adults collected in forested rural vs. urban habitats. The dashed red line is the fitted linear regression line (for detailed results, see Table S2).

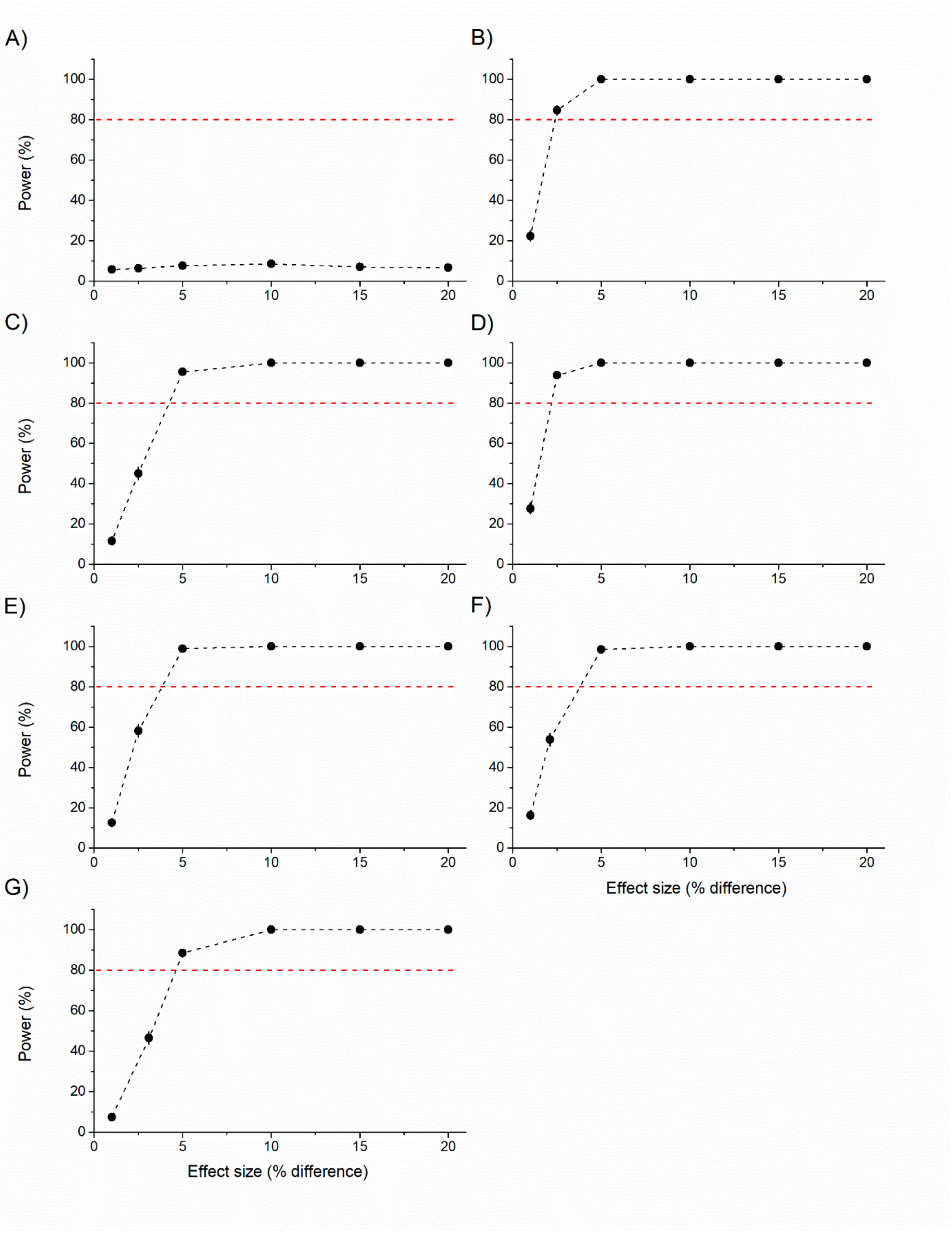

Figure S8. The statistical power (at $\alpha = 0.05$) of the linear mixed-effects models to detect 1-20% difference in the standardized pronotum volume (A), the standardized tibia and femur area of the front (B, C), middle (D, E), and hind legs (F, G) between urban and rural beetles with the original sample size (69 rural and 13 urban beetles). Red dotted lines indicate the generally accepted sufficient power limit (80%).

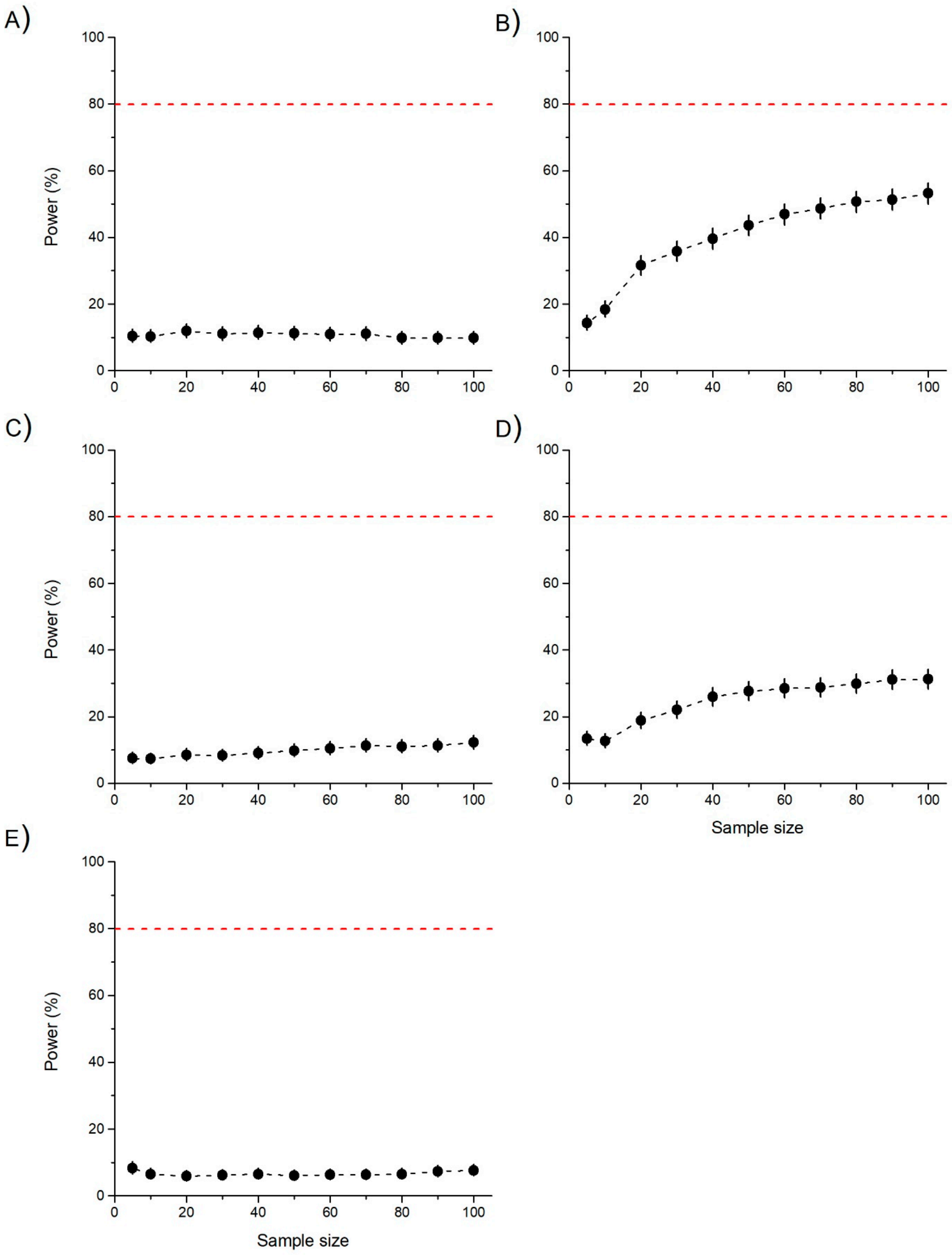

Figure S9. Statistical power (%) of linear mixed-effects models to detect the model-estimated differences in the standardized pronotum volume (A) and the standardized tibia and femur area of the front (B, C) and middle legs (D, E) between rural and urban beetles. Sample size represents the number of beetles per sex (female or male) per area (rural or urban). Red dotted lines indicate the generally accepted sufficient power limit (80%).
